# Healthcare professionals' perspectives on supporting individuals with NF1 during pregnancy and decision‐making processes

**DOI:** 10.1002/jgc4.70137

**Published:** 2025-11-11

**Authors:** Gamze Kaplan, Debbie M. Smith, Ming Wai Wan, Emma Burkitt‐Wright, Shruti Garg

**Affiliations:** ^1^ Division of Psychology and Mental Health, School of Health Sciences The University of Manchester Manchester UK; ^2^ Manchester Centre for Genomic Medicine Manchester University NHS Foundation Trust Manchester UK; ^3^ Division of Evolution and Genomic Sciences, School of Biological Sciences The University of Manchester Manchester UK; ^4^ Royal Manchester Children's Hospital Manchester University NHS Foundation Trust Manchester UK

**Keywords:** expectant parents, genetic counseling, NF parents, NF specialists, preimplantation genetic testing

## Abstract

Neurofibromatosis type 1 (NF1) is an autosomal dominant genetic condition characterized by highly variable presentation, making reproductive decision‐making and pregnancy care particularly complex. While previous research has focused largely on clinical outcomes, little is known about how healthcare professionals (HCPs) provide care and communicate with patients during this process. This qualitative study explores the views and experiences of HCPs in providing reproductive and pregnancy‐related care for individuals with NF1. Fifteen semi‐structured interviews were conducted with genetic counselors, NF specialist nurses, and clinical geneticists in the UK. Reflexive thematic analysis was used to analyze the data. HCPs described supporting informed reproductive choices as central to their role, but this was often complicated by the unpredictable nature of NF1 and varying levels of patient understanding. They emphasized the importance of discussing reproductive choices early, yet found it particularly difficult to offer clear guidance when patients had mild symptoms themselves or drew on diverse family experiences to interpret risk. These challenges were further compounded by systemic barriers, such as limited consultation time, lack of standardized communication tools, and insufficient training. This study highlights the need for more structured and consistent communication practices to support patients with NF1 during reproductive and pregnancy care. A simplified, context‐specific visual tool informed by the theoretical domains framework (TDF) may enhance counseling practice.

## 1 INTRODUCTION

Neurofibromatosis type 1 (NF1) is a rare genetic condition with an autosomal dominant inheritance pattern, caused by germline mutations in the NF1 tumor suppressor gene (Gutmann et al., 2017). It affects ~ 1 in 3000 individuals and presents with a wide spectrum of clinical features (Friedman, 2025). These include characteristic physical manifestations such as pigmentary lesions (café‐au‐lait macules and skinfold freckling), Lisch nodules and dermal neurofibromas. Additionally, NF1 is often associated with cognitive and behavioral challenges, including learning disabilities, and social and attention difficulties, which can significantly impact quality of life (Gutmann et al., 2017). However, the phenotypic severity of features varies widely between individuals, including in the same family, making it challenging to predict the clinical manifestation of NF1 (Kenborg et al., 2022).

The unpredictable nature of NF1, with its highly variable presentation, combined with the 50% likelihood of an affected parent passing the condition to their child, can introduce significant complexity to reproductive decision‐making for patients and their partners (Crawford et al., 2015; Rietman et al., 2018). The uncertainty surrounding how the condition might present in their children can be an important consideration for many families planning to have children (Crawford et al., 2015; Pepe et al., 2021). While family planning can already be a complex personal process, the added consideration of NF1 inheritance can bring extra challenges. In addition to genetic risks and uncertainties, pregnancy may present unique challenges for individuals with NF1, as women with the condition face an increased risk of obstetric complications, such as hypertension, preeclampsia, and preterm labor (Leppävirta et al., 2017; Radtke et al., 2020) and issues related to the growth of neurofibromas or plexiform tumors during pregnancy (Cesaretti et al., 2013). In such situations, access to genetic counseling and support may provide valuable guidance, helping individuals and families explore their options thoughtfully and make informed choices (Guo et al., 2023).

Various reproductive options are available to individuals with NF1, including natural conception, natural conception with prenatal diagnosis (PND) and the option to terminate an affected fetus, preimplantation genetic testing (PGT), adoption, the use of donor gametes or embryos, surrogacy, and the choice not to have children (NHS Commissioning Board, 2013). While all are valid decisions, we will focus specifically on natural conception with and without PND, and PGT, as these were topics of most frequent discussion during genetic counseling.

PGT, a procedure that involves testing embryos created through in vitro fertilization (IVF) for the genetic condition, with only unaffected embryos being transferred to the uterus (Geraedts & De Wert, 2009). In the UK, PGT is available through the publicly funded National Health Services (NHS) for individuals with a 10% or higher risk of conceiving a pregnancy affected by a serious genetic condition, including NF1. Specific eligibility criteria must be met for PGT: completion of genetic counseling through the local genetic service, the female partner being under 40 years of age with a body mass index (BMI) between 19 and 30, both partners being nonsmokers, and the couple or single parent not having an unaffected child, if a couple, being in a stable relationship for at least 1 year, and currently living at the same address (Guy's and St Thomas' NHS Foundation Trust, 2025). While PGT offers a valuable option for family planning, it involves a combination of hormonal treatments, surgical procedures, and the physical side effects associated with these interventions (Kostić et al., 2021). Even though the success rates are promising (Merker et al., 2015; Vernimmen et al., 2023), there is a possibility of failure at various stages.

Another option for expectant parents with NF1 is invasive prenatal diagnostics (PND), such as chorionic villus sampling (CVS) or amniocentesis. These tests can determine whether the fetus has inherited NF1, allowing parents the choice to terminate the pregnancy if the fetus is affected or to continue the pregnancy with the knowledge of the baby's NF1 status. However, while prenatal testing is possible when the causative NF1 variant has been identified in a parent, it cannot predict the severity of the condition in the child (Gutmann et al., 2017; Terzi et al., 2009).

These decisions might be accompanied by major emotional weight. Studies report feelings of guilt, anxiety, fear, and even grief, particularly in relation to the possibility of passing on the condition or making decisions around termination (Benjamin et al., 1993; Carrieri et al., 2016; Crawford et al., 2015; Gonzalez et al., 2025; Ponder et al., 1998). The volume and complexity of information involved in reproductive planning can feel overwhelming, especially when paired with uncertainty and time pressure. Gonzalez et al. (2025) found that many participants described feeling unprepared, confused, or unsupported when navigating these decisions, underscoring the need for comprehensive, accessible information and reassurance to help individuals and couples make informed decisions and cope with the emotional demands of the reproductive journey.

People with NF1 take diverse approaches to reproductive decisions, with some choosing to conceive naturally without pursuing genetic testing during pregnancy; others may view the likelihood of passing on NF1 more cautiously. Several factors may influence these decisions, including their personal experiences with NF1, concerns about miscarriage risks, the potential for uncertain or inconclusive results, and their attitudes toward termination (Di Mattei et al., 2021; Tzela et al., 2024). Cultural and religious beliefs, and their access to resources such as counseling or support groups can also play a significant role (Tzela et al., 2024; Zuckerman et al., 2020). Familiarity with the condition can also impact these choices. Individuals with a family history of NF1 are generally less likely to pursue prenatal testing compared with those with de novo variants, possibly reflecting greater personal acceptance of the condition's variability (Cesaretti et al., 2013). For healthcare professionals (HCPs), these diverse influences present challenges not only in delivering complex medical information but also in responding sensitively to varied patient values and expectations. Understanding how HCPs navigate these nuanced conversations is essential to support both the medical and emotional needs of parents, ensuring they feel informed about decisions throughout the process (Solem et al., 2020).

Currently in the NHS (2025), individuals with NF1 are typically referred by their primary care physicians – General Practitioners (GP) in the UK – or overseeing specialists to genetic counselors to discuss reproductive options and the likelihood of passing on the condition. During pregnancy, NHS website information on NF1 (2023) recommends that patients be cared for by an obstetrician with knowledge of NF1 or in consultation with an NF1 specialist, due to the known increased risk of complications.

While a few previous studies have provided valuable insight into the decision‐making experiences and needs of individuals living with NF1 (Benjamin et al., 1993; Carrieri et al., 2016; Crawford et al., 2015; Gonzalez et al., 2025; Ponder et al., 1998) there remains a major gap in understanding how HCPs approach the complexities of NF1 care. A recent review by Kalmantis et al. (2021), which synthesizes findings from 22 studies—including both retrospective cohort studies and case reports—focuses primarily on the medical risks of pregnancy in NF1 and supports the classification of these pregnancies as high‐risk, due to potential changes in symptoms and increased obstetric and perinatal complications. The review highlights that the focus of studies this far has been on outcomes rather than clinical perspectives.

Understanding HCP experiences is critical for informing clinical improvements, as they are central to balancing complex information delivery with supporting patient autonomy (Cesaretti et al., 2013; Garcia et al., 2022; Radtke et al., 2020). Evidence from other conditions illustrates how provider communication and views directly shape patient experiences. For instance, Kay et al. (2023) examined recurrence risk counseling for de novo mutations and found that this was a challenging process, particularly when couples sought complete reassurance. The authors concluded that implementing new strategies required additional training and counseling time. Similarly, Farrelly et al. (2012) observed that prenatal counseling for Down syndrome often emphasized the medical model of disability, with limited engagement with patient values, underlining the need for more holistic, patient‐centered discussions. In the context of indeterminate spinal muscular atrophy results, Spangenberg et al. (2025) described the emotional intensity and patient misunderstandings arising from time‐limited and complex consultations. Together, these findings demonstrate that technological advances alone do not guarantee effective clinical care; successful translation into practice requires addressing the practical, emotional, and systemic challenges faced by HCPs. Given the complexity and uncertainty associated with NF1, exploring the perspectives of HCPs is essential to identify barriers, support improvements in communication, and enhance patient‐centered service delivery.

This qualitative study aimed to address this knowledge gap by exploring the views and experiences of HCPs in providing reproductive and pregnancy‐related care for individuals with NF1. By focusing on the views of those involved in delivering care, this research aims to contribute to a more comprehensive understanding of how tailored, patient‐centered approaches can be developed to improve outcomes for individuals with NF1 and the services they receive.

## 2 METHODOLOGY

### 2.1 Design

A qualitative methodology was used to understand HCPs' views and experiences of providing care to expectant parents with NF1. Informed by a critical realist ontology (Bhaskar, 2020), this study places primary emphasis on the subjective accounts of HCPs regarding their work with expectant parents affected by NF1, while also recognizing that these perspectives are shaped by and connected to biological, clinical, and social realities. The approach acknowledges the interplay between individual experiences and the broader organizational and societal contexts in which care is delivered. The study was approved by the NHS Health Research Authority as an amendment to the existing ethics for the larger project EDEN‐P (reference 21/NW/0346).

### 2.2 Positionality

GK (she/her) is a *cis*‐gender, able‐bodied, Turkish, white researcher, and a nonnative English speaker. She is a PhD candidate in Psychology and Mental Health with a background in Education. Her work is guided by patient‐centered perspectives and informed by a neurodiversity approach, attending to structural inequalities while recognizing patients' strengths and agency. DMS is a health psychologist and mother. She has over 10 years of experience in qualitative research in pregnancy and has personal experience of living with a genetic condition. MWW is a British Chinese, English‐speaking academic in developmental psychology with 22 years' experience in parenting, parent–child dynamics, and neurodevelopmental research, including some prior knowledge of NF1. She teaches in mental health and holds a Divisional Equality, Diversity and Inclusion role, with prior qualitative work involving multidisciplinary HCPs. As a mother and developmental psychologist, MWW brings a reflexive awareness of power dynamics, equity, and child‐ and family‐centered values to her research. EBW is a consultant clinical geneticist with 19 years' experience in consulting with families with neurofibromatosis type I (NF1) and other genomic conditions and working within a comprehensive NF1 service in the NHS. Clinical genomic practice is underpinned by nondirective counseling, especially around reproductive issues, and multidisciplinary working. SG is a medical doctor of Asian Indian descent, trained in child psychiatry and currently working as a clinical academic. She has extensive experience supporting children and families affected by NF1, which informs her sensitivity to the medical and psychosocial complexities faced by those living with the condition. Her perspective is further shaped by her own lived experience of preeclampsia during pregnancy.

As an outsider to clinical genetics, GK conducted the interviews with no prior professional ties to participants, which encouraged more detailed sharing and reduced concerns about professional judgment. Analyses led by GK and DMS, with feedback from SG and EBW, both clinicians who work closely with genetic counselors, provided an insider perspective grounded in clinical practice and service realities.

### 2.3 Participants and recruitment

HCPs, including genetic counselors, doctors and specialist nurses who are working with people with NF1, were approached through the genetic services at Manchester Foundation Trust and by an NF charity. Potential participants were identified and referred by colleagues or service leads based on their professional experience with NF1. Since we recruited HCPs through referral, there was no risk of impostor participants; we did not include any methods to check identity. We set out to recruit 15 participants based on the past research experience of supervisors. Participants were eligible for interviews if they had experience supporting people with NF1, regardless of whether this support took place during pregnancy, as we wanted to capture broader interactions with patients, which could provide valuable insights and a more complete picture of their decision‐making and pregnancy experiences. The research team contacted potential participants via email to confirm eligibility and provide information about the study's aims and procedures. Participants were given the opportunity to ask questions, after which informed consent was obtained by email, and an online interview was scheduled. All but one person responded to the invitation; the nonresponder received three reminder emails. We had no prior contact or relationship with the participants before recruitment.

### 2.4 Data collection

Semi‐structured interviews were chosen for data collection due to their flexibility and ability to generate rich, in‐depth insights into sensitive topics (Braun & Clarke, 2013). A draft semi‐structured interview topic guide was created by GK, a doctoral student and DMS, a co‐investigator with extensive experience in qualitative research, informed by both previous literature and preliminary interviews with expectant parents with NF1. Key themes from the literature, such as communicating complex medical information, ethical considerations, interprofessional collaboration, and emerging reproductive options (Kay et al., 2023; Middleton et al., 2007; Parikh et al., 2023), were translated into topic areas. Insights from parent interviews, particularly around uncertainty, variability of experiences, and follow‐up care, shaped the prompt questions. This process ensured the guide was grounded in both existing evidence and lived experience. The topic guide was pilot‐tested with the first participant and reviewed by the supervisory team, including SG, DMS, and MWW. Only minor revisions were made based on feedback from the pilot, which led to rewording the final question to avoid assuming that HCPs perceived uncertainty among parents (see Table 1 for final version).

**TABLE 1:** Semi‐structured interview topic guide.

**Please tell us about your experience of supporting people with NF1 when they are pregnant or considering parenthood**. Prompt questions: Could you provide examples of effective strategies you've used to communicate complex medical information to patients with NF1 during their pregnancy/decision‐making? How do you inform patients about the existing risk of inheritance? How do you follow‐up with patients between appointments? Have you encountered any unique or unexpected challenges when caring for pregnant individuals with NF1? How did you address them? Can you describe a successful collaboration with other healthcare professionals in managing a patient with NF1 during pregnancy/decision‐making? What resources or support networks do you typically rely on when faced with complex cases of NF1 in pregnancy/decision‐making? Is there any training you're providing to expectant parents with NF1 or benefiting yourself? Are there any ethical or emotional considerations that you find particularly important when working with expectant parents with NF1? If any, how do you manage the uncertainties they feel?

GK conducted the semi‐structured interviews following the topic guide and used prompts to expand the points raised by participants. The interviewer had a patient‐centered perspective that informed the aim to explore HCPs' experiences to help improve the quality of care. During the interview, notes were taken, and a researcher's journal was maintained, reflecting on key insights, confusions, assumptions, analytic decisions, and reflexive considerations (Braun & Clarke, 2022). The interview was conducted using Zoom or Microsoft Teams, chosen for its ease of access and flexibility to accommodate HCPs' schedules, and audio‐recorded for later transcription.

### 2.5 Data analysis

Braun and Clarke's (2022) reflexive thematic analysis, chosen for its emphasis on researcher subjectivity, reflexivity, and active engagement with the data, guided an inductive latent‐level approach. The analysis began with familiarization, in which three stages—familiarization, coding, and theme generation—were first applied to two transcripts sequentially to build initial impressions. A supervisory meeting was held between DMS and GK to discuss ideas. Following this, both DMS and GK independently applied the same three stages to the entire dataset.

As coding progressed, NVivo 12 (2017) and ongoing note‐taking were used to support theme development, with attention to both semantic and latent meanings (step 4). Two additional supervisory meetings were held to refine, relabel, and summarize the themes (step 5). The process was interactive and iterative, informed by reflective journaling and discussions to ensure rigor and reflexivity. The study reached information power, capturing adequate depth and complexity across themes in relation to the study aim (Malterud et al., 2016). The final write‐up of the findings was carried out by GK (step 6).

### 2.6 Mapping the results

Following inductive reflexive thematic analysis, the findings were mapped onto the theoretical domains framework (TDF) and further informed by uncertainty communication theories. This step was undertaken because our themes showed close alignment with the TDF, and we aimed to translate them into a concise, accessible tool for HCPs, prioritizing ease of use within their busy schedules. The definitions and contextual guidance provided by Atkins et al. (2017) supported the process. The mapping of the themes onto the framework is presented in Appendix A.

## 3 RESULTS

**FIGURE 1:**
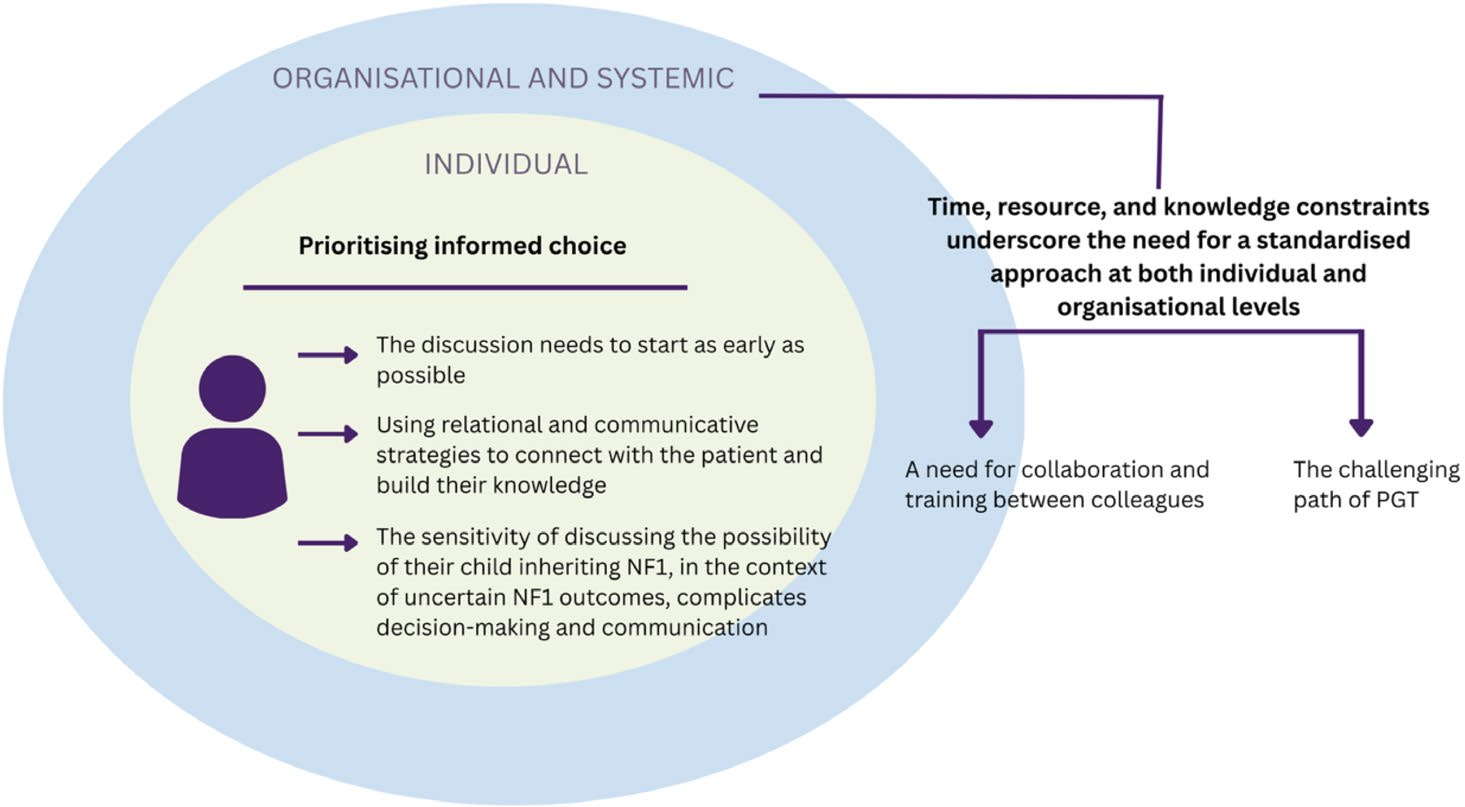
Reached themes and subthemes through reflexive thematic analysis.

Fifteen interviews were conducted between February and May 2024 with HCPs, including NF specialist nurses, genetic counselors, and clinical geneticists from across England and Wales, with available information on years of experience for seven participants ranging from 3 months to 35 years (M = 8.39 years, SD = 12.20), with one of them being male, interviews lasted between 21 and 48 min (M = 33.8 min, SD = 8.01). The number of professionals in each role is not reported to protect confidentiality. Based on the interviews, two themes were generated: *prioritizing informed choice* and *time, resource, and knowledge constraints underscore the need for standardized processes at both individual and organizational levels*. These themes will be presented with their subthemes and direct quotes from the participants to support the related statements (Figure 1).

### 3.1 Theme 1 – Prioritizing informed choice

All HCPs emphasized the importance of informed decision‐making to prevent unplanned pregnancies. They shared various stories about uninformed patients or cases in which ambiguous language and misinformation led to confusion. Despite prioritizing the delivery of early information, they reported that some NF1 patients had gaps in their understanding while others were well‐informed about their options. Although many patients were aware of the 50% inheritance patterns of NF1, there were still some misconceptions and gaps in the understanding of the genetic basis or pregnancy complications, which influenced their decision‐making and anxiety level around the idea of having a child. Three subthemes, namely, “The discussion needs to start as early as possible”, “Using relational and communicative strategies to connect with the patient and build their knowledge”, “The sensitivity of discussing the possibility of their child inheriting NF1, in the context of uncertain NF1 outcomes, complicates decision‐making and communication” further expand this theme, reflecting the key aspects of providing information about the reproductive options and guidance on pregnancy to make an informed choice.

Sometimes people have things a little bit wrong. They would, they might sort of say, for example, that they're like mother's side, so they always knew that they [the child] would have it. That kind of thing, and I think you don't want to go in and challenge that. (HCP‐12)

#### 3.1.1 Subtheme 1 – The discussion needs to start as early as possible

All HCPs prioritized providing early information about the dominant inheritance patterns and pregnancy options to prevent unplanned pregnancies and prepare for future planning. This information is typically shared during the transition from pediatric to adult services around the age of 16, during prepregnancy counseling, or, in some cases, HCPs inform parents of children with NF1 about their child's future reproductive options while the child is still young. While early information was perceived as crucial for preventing unplanned pregnancies, it may not be remembered by patients or given the necessary attention over time due to shifting life priorities.

But while they are with us, we try and get that genetics meeting in place. But you know what it is like, it is when they are young 16, 17, you know they may not take much notice of the meeting and they might not take all in. (HCP‐4)

Some HCPs underscored the importance of reaching out to wider family networks following the diagnosis of a child in the family, not only to support partners but also to identify undiagnosed individuals across generations who may have previously misunderstood or minimized their condition, which can help ensure that families are better informed and able to engage with relevant information and options earlier in life. While this is not limited to pregnancy, early identification can be particularly valuable for those who may be planning a family, enabling earlier access to genetic counseling and support.

Families find out often the child will be diagnosed, and then we diagnose one of the parents, one of the grandparents, aunties and uncles, and they would say, “Well, all we've ever been told is all they just birthmarks and that's it”. (HCP‐8)

#### 3.1.2 Subtheme 2 – Using relational and communicative strategies to connect with the patient and build their knowledge

Some HCPs described how they used relational and communicative strategies not only to share information, but to actively build trust and support patients' understanding of heritability and reproductive options over time. Regular appointments were found to be beneficial for building patients' knowledge and encouraging them to ask questions within the services provided. Giving patients time and space, being there and listening to them, and letting them know that, regardless of what they choose, there is support that they can reach whenever they need, were seen as key aspects of a well‐designed appointment. Rather than relying solely on verbal explanations, some professionals also used tools such as personalized letters, diagrams, and gestures to reinforce understanding, recognizing that different formats could help patients process complex information more effectively, thinking that “it adds value to what [they] already discussed in the clinic (HCP‐7)”. These strategies were underpinned by a commitment to sensitive, patient‐centered communication. Most participants emphasized the importance of tailoring information to individual needs, being honest and clear, and fostering a sense of partnership. For some, this meant consciously shifting away from a traditional, hierarchical model of care toward a more collaborative dynamic, encouraging patients to share their own experiences and knowledge as part of the conversation.

Actually I try to get onto the same level, so it's a shared experience … you tell me what you know about the condition, you tell me how it's affected you and it's more of a conversation rather than a teaching and learning experience. And I think that helps build rapport and builds trust in me. (HCP‐11)

#### 3.1.3 Subtheme 3 – The sensitivity of discussing the possibility of their child inheriting NF1, in the context of uncertain NF1 outcomes, complicates decision‐making and communication

Most HCPs described how the unpredictable nature of NF1 complicates reproductive decision‐making and communication around it, particularly around prenatal testing. Although prenatal testing can confirm the presence of an *NF1* variant in the fertilized egg, it does not provide insight into the severity of the condition in children. Some described how parents struggled with the decision to terminate a pregnancy as it is not possible to know how severely the child would be affected, especially when the outcome for the child could be relatively mild. The inability to provide clear outcomes contributed to a sense of emotional burden for both parents and clinicians.

[from a patient's perspective] What if I end a pregnancy for an affected baby, but actually that child was only going to be mildly affected — potentially no symptoms in childhood, maybe just a few café‐au‐lait spots… That's really an ethically quite challenging discussion to have. (HCP‐11)

These conversations were more complex when NF1 was already present in the parent or another family member. Some HCPs shared that discussing reproductive options in such contexts could feel deeply personal, raising the possibility of avoiding transmission could feel like a judgment on their own or their child's lives, especially when individuals had lived with well‐managed NF1 for many years.

But ensuring you have a child without NF1 is almost like saying to them, well, we make sure somebody like you isn't born, which is really difficult and some parents find that incredibly difficult to understand. (HCP‐2)

Most HCPs also observed that parents' decisions were influenced by their own or family members' experiences when making decisions. One HCP remarked that, in some cases, “people do think because they're mildly affected they think the baby is gonna be mildly affected (HCP‐13)”, which required careful communication about the condition's variability. They considered the perinatal period as particularly challenging for families, regardless of the decisions made, with concerns extending beyond genetic risk to include tumor growth, pregnancy complications, and long‐term fears about the child's appearance, relationships, and future reproductive choices.

it was just more the unknown and she [patient] was quite distressed that how many neurofibromas she got during this pregnancy, and you know just after that she was really distressed, it caused her a lot of anxiety. (HCP‐1)

She[patient] was worried about her daughter's future, … marriage, and how she would look. (HCP‐13)

Most HCPs noted a general lack of parents' awareness about PGT and mentioned that they inform patients about this option when appropriate. While some HCPs mentioned that patients often show interest in PGT to make the pregnancy less anxiety‐provoking, others expressed that patients with NF1 tend to request PGT less often than those with other genetic conditions, potentially reflecting a perception among patients that NF1 is medically less severe:

The number of people that request some kind of prenatal diagnosis is probably not as high as we've seen other genetic conditions … I think they just thought, well, it's more of a cosmetic condition and therefore that's what is going to be passed on to children. (HCP‐8)

### 3.2 Theme 2 – Time, resource, and knowledge constraints underscore the need for a standardized approach at both individual and organizational levels

Most HCPs identified systemic issues that shaped how they provided care to families with NF1. These challenges reflected broader issues in healthcare systems, including limited time to dedicate to NF1‐specific care and longer waiting lists, especially following the COVID‐19 pandemic. While all HCPs expressed a desire to support families, they often worked within systems that limited their ability to provide required care. In response to time constraints, communication often shifted from in‐person appointments to phone calls and emails, with families encouraged to get in touch if needed.

Because of just the volume of work, we kind of trust that if there is any concern they will make their way back to us. So actually, those families I would tend not to kind of again actively follow‐up unless they came back to us in another pregnancy. (HCP‐12)

A few HCPs also described inconsistencies in access to services, particularly psychological support. This regional disparity in service delivery was detailed by HCP‐4 “if they do not have another diagnosis, another disability diagnosis like ADHD, learning disability or autism, our psychology team won't accept them. … But I do believe the [location] team have a specific psychology team that support NF children.” Funding limitations were also noted by some. For instance, while additional payments are available for GPs working with certain patient groups, there are no such incentives for NF1 patients, which may lead to a lack of perceived responsibility. Some HCPs highlighted the need for a standardized approach to ensure all patients receive consistent information about reproductive options, regardless of where they live, which service they access, or which professional they see, noting that “I don't think there's any tick‐box guideline to make sure that every healthcare professional is actually doing that” (HCP‐9). While these issues encompass the whole procedure, two specific subthemes focused on “A need for collaboration and training between colleagues” and “The challenging path of PGT”.

#### 3.2.1 Subtheme 1 – A need for collaboration and training between colleagues

All HCPs described collaboration with colleagues as essential for delivering effective care and developing confidence when managing complex NF1 cases. In the absence of condition‐specific training, many professionals relied on informal learning from more experienced colleagues. Senior team members were often viewed as key sources of knowledge, and observation was highlighted as a valuable way to learn how to communicate risk and support decision‐making.

I've learned quite a lot of the sort of counselling that I do about NF1 as a condition and what the risks are is very much based on things I've heard her saying to patients … I'm really kind of basing that off literature, and from working with her really. (HCP‐14).

MDTs were frequently discussed as aiding collaboration, offering opportunities to bring together diverse professional perspectives and adopt a more holistic approach to care. These were described as a setting where HCPs could raise concerns, seek guidance, and share responsibility when cases felt outside their expertise.

So any concerns I have from my end, I take it to them at the MDT and ask for advice, and you know, obviously, if I feel it is out of my capabilities, they will chat to the patients. (HCP‐6)

Despite the benefits of collaboration, a lack of specialist knowledge about NF1 among some HCPs was described as a challenge, particularly in communication with GPs. Some participants reported difficulties when engaging with GPs who lacked awareness of the condition or had limited time to respond to its complexity: “the GPs just do not understand the condition… so little knowledge around NF1” (HCP‐1), although they are valued for their role in facilitating rapid referrals. Some HCPs signposted families to external resources, such as charities for accurate information and nondirective support, and social media groups for peer connection, while noting that some online content could be distressing or misleading.

#### 3.2.2 Subtheme 2 – Challenging path of PGT

PGT can be a complex process for families considering it. Although it can offer an option to avoid passing on the condition to their child, the availability of government funding in the UK is limited by specific eligibility criteria. These include age restrictions and whether the couple already has children, which can make some families ineligible for funded treatment. A few HCPs reflected on their own limited awareness of these criteria, which sometimes made them feel uncertain when discussing options with patients.

I don't know this is a fact but I've heard it said that you know patients to be referred for the PGT programme they have to be a certain age. (HCP‐9)

The process was described by some HCPs as challenging and emotionally demanding for both partners, particularly for women. In some cases, the woman was not present during the clinical consultation, often because the male partner was the one affected by NF1 and initially sought information or made decisions alone. Some HCPs noted that they made additional efforts to involve female partners, ensuring they received accurate information and could participate in fully informed decision‐making.

Well, his wife was not with him at clinic…So then I just offered if she did want to discuss this, but he said, oh, I don't care about that. I'm just going to take 50‐50 chance, knowing the risks. (HCP‐6)

### 3.3 Mapping the results to theoretical frameworks

**FIGURE 2:**
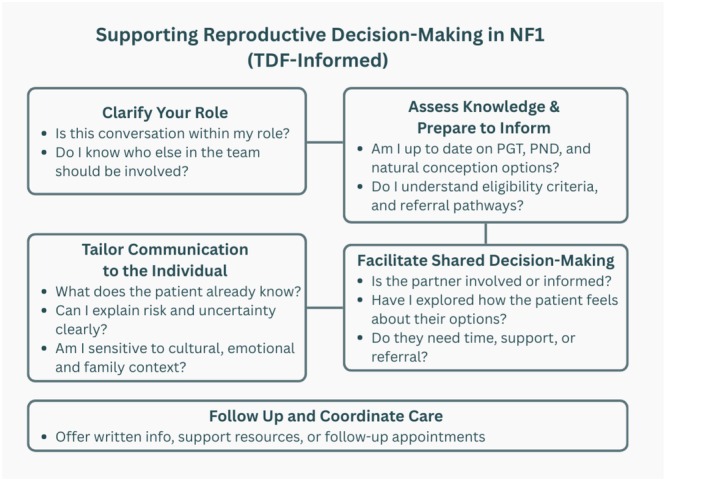
A context‐specific tool designed to support reproductive decision‐making in individuals with NF1, informed by data in this study, the theoretical domains framework and uncertainty communication theories.

Following the inductive analysis, the themes were mapped onto the TDF and uncertainty communication theories to explore their practical implications. This mapping highlighted eight domains most relevant to reproductive care in NF1 (knowledge, skills, social/professional role, beliefs about capabilities, environmental context, social influences, emotion, and behavioral regulation). These were grouped into cognitive, affective, and behavioral aspects of uncertainty communication. This process informed the development of a simplified, context‐specific communication tool, designed to support HCPs in holding more consistent conversations about reproductive options (Figure 2, see Appendix A for full details of the mapping).

## 4 DISCUSSION

This study explores the views and experiences of HCPs, including genetic counselors, doctors and specialist nurses, in providing reproductive and pregnancy‐related care for individuals with NF1 in the UK. Using a reflexive thematic analysis approach, the findings showed that supporting informed reproductive choices was central to HCPs' practice, yet often complicated by the variable nature of NF1, which made it difficult to offer clear guidance during reproductive counseling. This uncertainty was described as especially difficult when patients had mild symptoms themselves or were drawing on varied family experiences to interpret risk. Even when patients were familiar with NF1, differences in understanding added to the complexity, further compounded by systemic issues such as time constraints and the absence of standardized communication processes.

HCPs emphasized that informed reproductive decision‐making is central to the care of individuals with NF1 and should begin as early as possible. However, this process is inherently complex, and the variability of NF1 further contributes to this uncertainty. For both patients and HCPs, the inability to predict how the condition may present in a future child complicates decision‐making (Pepe et al., 2021). This uncertainty often creates emotional dilemmas for expectant parents to balance feelings of fear, hope, and concern for their child's future. As Brashers et al. (2000) explain, people respond to uncertainty in different ways; sometimes it causes anxiety or worry, but it can also bring hope. When the perceived threat of information is high, some may seek further information in order to maintain a level of uncertainty that feels more manageable. Thus, managing uncertainty does not always mean using strategies to reduce it, but rather supporting individuals in how they interpret and cope with it. This underscores the importance of counseling patients and supporting them emotionally. These needs for care and counseling were also emphasized in a study by Gonzalez et al. (2025) from a patient perspective in an Australian participant group as enablers of reproductive decision‐making. Dean and Street (2015) highlight communication strategies to support patients in managing uncertainty that address cognitive, emotional, and behavioral domains. Importantly, HCPs noted that patients come to consultations with varying levels of knowledge; some are well‐informed through personal experience or prior research, while others have little or no background understanding. To avoid making assumptions about what patients know, it can be important to actively explore their understanding at the beginning of the consultation. Additionally, their perception of uncertainty may change over time, and to balance it, patients may need to add to their understanding (Bylund et al., 2012). Therefore, counseling for informed decision‐making is not simply about providing information at a single point in time, but about ensuring that it is understood and can be retained over time.

The timing and delivery of communication emerged as critical aspects of care, with HCPs emphasizing the importance of introducing reproductive information early and revisiting it throughout the patient's life. In current UK practice, NF1 care is typically transferred to GPs after the age of 16, unless specialist input is needed (Vassallo, 2019). Although many HCPs reported raising reproductive choices and decision‐making during the transition period and at any point of contact with patients, there is no structured approach or formal training in place to guide these discussions. They often stated a reliance on experiential learning, informal mentoring, or observation, which can lead to inconsistencies in how information is communicated and interpreted. This highlights the need for a more consistent and theory‐informed approach to communication. The TDF offered a useful framework, providing insight into the cognitive, emotional, social, and environmental influences on healthcare practice (Atkins et al., 2017). Aligned with Dean and Street's (2015) communication model, the TDF could inform more reflective and consistent genetic counseling practices. However, its practical application may be limited by time constraints and the need for specialist training (Phillips et al., 2015).

A more feasible alternative could involve a simplified, context‐specific TDF‐based tool to support conversations around reproductive options and related decision‐making. The visual tool developed in this study selectively applied domains from the TDF, based on relevance to our results. This targeted approach is consistent with guidance on TDF use in implementation research, ensuring the tool remains grounded in both behavioral theory and real‐world practice (Atkins et al., 2017; Cane et al., 2012). Systematic reviews have also demonstrated the adaptability of the TDF across diverse healthcare contexts, such as prescribing guideline adherence (Paksaite et al., 2021), medicine risk communication (Alharbi et al., 2024), and referral practices in genetics (Morrow et al., 2021). Reviews further highlight its value for mapping determinants of behavior change in primary care and broader clinical implementation (Birken et al., 2017; Dyson & Cowdell, 2021; Mather et al., 2022). Our study tries to extend this literature into an implementation context by developing and illustrating a simplified, context‐specific adaptation of the TDF to support reproductive decision‐making discussions in NF1 care. In practice, this could be complemented by MDT input, particularly when clinicians feel underprepared or uncertain about how to approach complex or sensitive discussions. In such cases, MDTs offer a valuable source of shared expertise and can help ensure that patients receive coordinated and well‐informed support.

Our findings underscore a gap in NF1‐related practice among GPs in NF1 practice, echoing Evans et al. (2020), who noted that many GPs feel ill‐equipped to manage NF1 patient care and emphasized the need for concise, relevant, and primary care‐specific resources. Building on this, Evans et al. (2025) proposed a structured, primary care—focused pathway for NF1, including early preconception referral to Clinical Genetics, routine antenatal monitoring, and a comprehensive transition review in adolescence covering reproductive options and wider support needs. This framework offers a pragmatic approach for GPs and nonspecialists to engage more consistently in reproductive discussions given constraints of limited genetic service capacity. However, while Evans et al. (2025) acknowledged the potential role of rare disease specialist nurses as care coordinators, this role was not universally embedded in the healthcare system. Our results suggest that explicitly integrating NF specialist nurses could provide continuity and relational depth, while standardized communication tools, MDT input for complex cases, and signposting to reliable resources could further strengthen care pathways. Embedding these components may help minimize variability and ensure patients receive coordinated, accurate, and sensitive guidance across care settings.

### 4.1 Limitations

While some HCP experiences may be similar in other Western healthcare systems, these findings should be interpreted within the specific context of the UK's NHS and its associated policies. The findings of this study are based on the HCPs' views and experiences; thus, it does not reflect the direct experience of patients living with NF1 or navigating reproductive choices themselves. Furthermore, we did not set a minimum experience threshold for working with individuals with NF1, meaning some HCPs had limited practice in pregnancy, family planning, or reproductive decision‐making in this group, potentially limiting the breadth and depth of perspectives captured.

### 4.2 Implications for research and practice

The findings of this study have policy implications, particularly in highlighting the need for ongoing training and a more structured approach to reproductive counseling within NF1 care. While Figure 2 presents a brief, TDF‐based guideline to support discussions around reproductive options, this adapted framework has not yet been evaluated. Future research should investigate the feasibility, applicability, and effectiveness of implementing a shortened version of the TDF in clinical practice. It would also be valuable to conduct similar studies in other countries to explore how national policies and healthcare structures influence current practices and to enable more comparative or cross‐cultural insights. Additionally, future research could explore patients' perspectives to deepen clinical understanding of reproductive decision‐making and pregnancy, particularly through comparison with healthcare professionals' experiences. Finally, given that HCPs in our study expressed uncertainty about PGT eligibility criteria, closer collaboration with fertility specialists, potentially through their inclusion in MDT discussions should be considered to ensure patients receive accurate and timely guidance on reproductive options.

## 5 CONCLUSION

To the best of our knowledge, this is the first study to explore HCPs' views and experiences of providing reproductive and pregnancy‐related care for individuals with NF1, both within the UK and in an international context. The findings highlight the need for a more structured approach to service provision, while emphasizing the importance of supporting informed choice through early discussions and revisiting information throughout the care pathway.

## AUTHOR CONTRIBUTIONS

GK led the conceptualization and design of the study, managed recruitment and communication, conducted the interviews, analyzed the data, and drafted the manuscript. DMS contributed to the conceptualization, supervised data collection and analysis, and provided critical feedback on the manuscript. MWW was involved in the conceptualization and contributed critical feedback. EBW supported recruitment by promoting the study to professionals and provided critical comments on the manuscript. SG provided primary supervision and contributed to the conceptualization, design, and recruitment, as well as offering critical feedback on the manuscript. All authors reviewed and approved the final version and agree to be accountable for all aspects of the work, ensuring that questions related to the accuracy or integrity of any part are appropriately investigated and resolved.

## FUNDING INFORMATION

This research was supported by the NIHR Manchester Biomedical Research Centre (NIHR203308). The views expressed are those of the author(s) and not necessarily those of the NIHR or the Department of Health and Social Care. Gamze Kaplan is a PhD student, and their study was supported by the Republic of Türkiye Ministry of National Education.

## CONFLICT OF INTEREST STATEMENT

GK, MWW, DS, EBW, and SG have no conflict of interest to disclose.

## ETHICS STATEMENT

Human Studies and Informed Consent: This study was approved by the NHS Health Research Authority (reference 21/NW/0346). All procedures were conducted in accordance with the approved protocol, relevant ethical standards, and the principles of the Helsinki Declaration (as amended in 2024). No identifying information was used in this study; all data were anonymized and stored securely. All participants provided written informed consent.

Animal studies: No animal studies were conducted as part of this research.

## Data Availability

The data that support the findings of this study are available from the corresponding author, SG, upon reasonable request.

## APPENDIX A The mapping of the themes onto the framework, which shows the underpinnings of the tool.

**Table:**

TDF domain	Do the themes from this study map onto the framework components?	Uncertainty communication theories	Relevance to findings	How it is used in the framework
1. Knowledge	Yes	*Cognitive*: Clarifying what is known/unknown helps reduce cognitive uncertainty	HCPs lacked clarity on PGT/PND eligibility, referral pathways, and available services	Step 2: *Assess Knowledge & Prepare to Inform*
2. Skills	Yes	*Affective*: Demonstrating empathy, adjusting tone, and managing emotional uncertainty	HCPs relied on observations to build counseling and communication skills.	Step 3: *Tailor Communication to the Individual*
3. Social/professional role and identity	Yes	*Behavioral*: Clear role identification reduces ambiguity in information delivery and support coordination	Role ambiguity, unclear responsibility for initiating reproductive discussions.	Step 1: *Clarify Your Role*
4. Beliefs about capabilities	Yes	*Affective*: Modeling vulnerability and shared uncertainty build trust in difficult conversations	Some HCPs concerned about discussing emotionally or ethically difficult topics	Step 3–4: Support for using team or referral when unsure
5. Optimism	No	Not applicable	No discussion of hopeful or pessimistic outcome expectations.	Not included.
6. Beliefs about consequences	Yes	*Affective*: Sensitivity to how patients perceive risk and moral judgment enhances trust	HCPs feared causing distress or being seen as judgmental about termination or severity	Step 3: *Tailor Communication*
7. Reinforcement	No	Not applicable	No reward or feedback systems described	Not included
8. Intentions	No	Not applicable	No explicit mention of forming intentions to change or improve practice	Not included
9. Goals	No	Not applicable	No goal‐directed action or planning behavior described	Not included
10. Memory, attention, and decision processes	No	Not applicable	No issues of recall or attention overload reported	Not included
11. Environmental context and resources	Yes	*Behavioral*: Providing clear pathways and resources helps patients manage practical uncertainty	Barriers included time constraints, regional disparities, lack of NF1‐specific clinics	Step 2 and Step 5: Prompts to coordinate resources and offer referrals
12. Social influences	Yes	*Behavioral*: Social support and shared expertise reduce isolation and reinforce confidence in uncertainty contexts	Peer learning, informal mentorship, MDTs, and collaborative discussions were central	Step 5: *Coordinate Care and Follow‐Up*
13. Emotion	Yes	*Affective*: Responding to emotional uncertainty with empathy strengthens therapeutic relationships	Emotionally intense conversations were common, gendered dynamics influenced communication	Step 3 and Step 4: Emphasizes empathy, partner inclusion, and validation
14. Behavioral regulation	Yes	*Behavioral*: Structure and continuity reduce uncertainty in fragmented systems	Some HCPs used structured approaches to ensure follow‐up.	Step 5: Use of structured care plans
